# Psychometric properties of the readiness for return to work scale in occupational rehabilitation in South Korea

**DOI:** 10.1186/s12889-022-14948-2

**Published:** 2023-01-06

**Authors:** Jeong-Eun Lee, Soohyun Kim, Gain Shin, Su Bin Yoo, Ja-Ho Leigh

**Affiliations:** 1 Department of Rehabilitation Medicine, Seoul National University Hospital, Seoul National University College of Medicine, Seoul, Republic of Korea; 2 Coresearch Coop, Seoul, Republic of Korea; 3 Department of Rehabilitation Medicine, Korea Workers Compensation and Welfare Service Incheon Hospital, Incheon, Republic of Korea; 4 Interdisciplinary Graduate Program in Social Welfare Policy, Yonsei University, Seoul, Republic of Korea; 5 National Traffic Injury Rehabilitation Research Institute, National Traffic Injury Rehabilitation Hospital, Yangpyeong, Gyeonggi-do Republic of Korea; 6 Clinical Research Center, Inje University Ilsan Paik Hospital, Goyang, Gyeonggi-do, Republic of Korea; 7 Institute of Health Policy and Management, Medical Research Center, Seoul National University, Seoul, Republic of Korea

**Keywords:** Motivation, Musculoskeletal, Readiness, Return to work, Validation

## Abstract

**Background:**

This study validated the Korean version of the Readiness to Return to Work (RRTW) scale, as an assessment measure, following a musculoskeletal, work-related injury and as a measure of following return to work.

**Methods:**

The participants of this study were workers with experience in rehabilitation programs at the Workers’ Compensation and Welfare Service (KCOMWEL) Hospital in Korea. Factor analyses were employed to ensure the validity and reliability of the RRTW scale in claimants who were in treatment without working (the not-working group) or who had already returned to work (the working group). To test structural validity, we analyzed exploratory factor analysis (EFA) respectively for the not working group (exploratory factor analysis (EFA) (*n* = 200), confirmatory factor analysis (CFA) (*n* = 109), and the working group (*n* = 123). To verify concurrent validity (multidimensional and assignment approach), the variables that were identified as relevant variables in previous studies were analyzed.

**Results:**

The not working group EFA, as shown in the original scale, had four dimensions, and one item was deleted: (1) Precontemplation (PC), (2) Contemplation (C), (3) Prepared for Action-Self-evaluative (PAS), and (4) Prepared for Action-Behavioral (PAB). The CFA revealed that a good model fit and reliability were suitable. Regarding the working group of EFA, it appeared in two dimensions as in the original scale, one item was modified from the UM scale to the PM scale, and the reliability was appropriate. Concurrent validity was satisfied based on the correlation between the RRTW factor and related variables.

**Conclusions:**

RRTW in the Korean version of the instrument was similar to those reported for the original scale, indicating that it may be used in research and clinical settings.

**Supplementary Information:**

The online version contains supplementary material available at 10.1186/s12889-022-14948-2.

## Background

Return to work (RTW) following an injury is a dynamic, evolving process and involves complex physical, psychological, and social factors [1–4]. Studies have identified workers’ own perceptions and motivational factors, such as expectations of recovery and RTW, or self-efficacy, as important common factors affecting RTW [4, 5] or sustainable RTW [6].

The readiness for change model, empirically supported for smoking cessation, weight control, or pain management, emphasizes motivational factors in facilitating and maintaining behavior change and the importance of stage-specific interventions [7]. Based on the readiness for change model, Franche and Krause developed the readiness for return to work (RRTW) model [7]. According to this model, returning to work was regarded as a behavior change and individual progress through stages of change towards RTW. They also developed the RRTW scale, a 22-item self-report measure, to identify workers in need of intervention, and confirmed its validity and reliability on a sample of injured workers with musculoskeletal disorders (MSK) [8]. They identified four stages in a sample of not-working group: Pre-contemplation (PC), Contemplation (C), Preparation for action-self-evaluation (PAS), and Preparation for action-behavioral (PAB), and two stages in a sample of working group: uncertain maintenance (UM) and proactive maintenance (PM), by factor analysis. Concurrent validity was confirmed as overall improvement was found in constructs related to depressive symptoms, fear-avoidance, pain, and general health, from less advanced to more advanced stages of readiness [8].

According to previous studies, exploratory factor analysis (EFA) and confirmatory factor analysis (CFA) were used to confirm the validity of both the RRTW scale and the scale model (see Additional File 1).

Validity tests of the RRTW scale have been conducted in different samples, settings, and countries; however, they did not show consistent psychometric properties for the not working scale. In a Canadian study of outpatients (MSK disorders: joint disorder, sprain/strain, fracture, other), the PC stage was not identified in the not-working scale [9]. In a Norwegian study with participants who had MSK or mental and behavioral disorders, only two factors (uncertainty and inability) were found in the scale for not-working [10]. Moreover, Stapelfeldt et al.’s study of the Danish version could neither confirm the factor structure of the original model nor the Norwegian model in confirmatory factor analysis [11]. A cross-cultural translation and adaptation of the RRTW scale for German patients in psychosomatic rehabilitation [12] and for Dutch cancer survivors [13] were also tested. Further research is needed on the psychometric properties of the RRTW scale and clinical usefulness for tailored intervention.

In Korea, an RTW support program is operated for injured workers who filed an accepted claim with the Korean worker’s compensation system. The program does not include motivational intervention but focuses primarily on hardening physical work capacity. To develop and provide a tailored intervention program for RTW and work maintenance, measuring an individual’s readiness for return to work is the most crucial first step.

## Objectives

The objective of the study was to validate the Korean version of the RRTW scale, as an assessment measure, following a musculoskeletal, work-related injury and as a measure of following return to work. First, we performed a cross-cultural translation of the RRTW scale into Korean and adapted the Korean version of the RRTW scale among Korean workers with work-related muscular-skeletal injuries. Next, in order to test structural validity, we conducted an exploratory and confirmatory factor analysis and examined concurrent validity using a multidimensional and a stage allocation approach.

## Methods

### The translation process

Forward- and backward-translation procedures were performed for the Korean version of RRTW. The original English version of the scale was translated into Korean after receiving permission from Renee-Lousie Franche, a developer of the RRTW scale. Two Korean versions were created: one by a professional bilingual translator and another by a bilingual rehabilitative medicine doctor. The two Korean translations were then unified by the authors after discussing conceptual equivalence and cultural differences. The Korean scale was reviewed by a Korean language and literature PhD candidate; then, it was translated back into English by a different professional translator who was unfamiliar with the original English version. The back-translation version was reviewed by the authors and Renee-Lousie Franche. Reflecting on Franche’s point, too definitive expression (item a4), omission of emphasis expression (item a13), and nuance differences (item b1, 2, and 7; stay at work) in the back-translation version were corrected. One item was adapted for the Korean context; that is, we added “or at the hospital” to item a5 (You have been increasing activities at home to build up strength to go back to work). As the ratio of inpatients and long-term patients with severe musculoskeletal injury is high in Korea Workers Compensation Insurance System, we attempted to cover both inpatients and outpatients.

As for the translated item, an individual survey was conducted on four rehabilitation experts, including rehabilitation doctors, physical therapists, and occupational therapists.

The understanding of items was reviewed for three patients visiting the industrial accident hospital to confirm the appropriateness and difficulty of the item.

### Design and participants

The study participants were workers who filed an accepted claim with the Korean worker’s compensation system following a work-related musculoskeletal injury. Workers were eligible to participate in the study if, as a claimant, they were: aged between 18 to 65 years at the time of participation; absent from work owing to a musculoskeletal injury ranging from fracture to amputation; were receiving or had received treatment, such as a subacute intensive rehabilitation program, tailored exercise program, or work-hardening program, and the treatment period had elapsed for more than six months owing to the above treatment procedures.

By setting these inclusion criteria, we attempted to include those who had a gap from work for more than three months owing to severe musculoskeletal injuries. Data for this study were obtained from two different sources: (1) cross-sectional survey data to investigate the construct and concurrent validity of the RRTW scale, and (2) the retrospective medical record data from patients’ Functional Capacity Evaluation (FCE) in Incheon Korea Worker’s Compensation & Welfare Service (KCOMWEL) hospital.

A cross-sectional survey procedure performed in studies involving human participants was in accordance with the ethical standards of the Institutional Review Board of Seoul National University Hospital (IRB No.1607–044-744/ 1902–109-1012). Moreover, this study was conducted after explaining the study to the participants and obtaining their consent to participate in it. All procedures were in accordance with the ethical standards of the responsible committee on human experimentation (institutional and national) and with the Helsinki Declaration of 1975, as revised in 2000(5). Additionally, approval was granted by the Ethics Committee of the Institutional Review Board of Seoul National University Hospital (IRB No.1607–044-744/ 1902–109-1012).

### Data collection

A cross-sectional survey was performed on claimants who were in treatment without working or who had already returned to work. They were invited by rehabilitation medicine doctors or physical therapists at KCOMWEL hospitals. The contact address of those who agreed to participate in the study was passed on to the authors, and the survey interviews were conducted by the authors and trained social work students. The participants were informed about the study, and they read the explanation of the study for a sufficient amount of time. The survey began when they agreed to participate in the study. They received $45 for their participation in the study. Face-to-face interviews were conducted between September 2016 and December 2018. A total of 202 participants in the not-working group and 72 in the working group completed the survey; however, two participants whose duration of work disability was over five years (too long) were excluded from the not-working group. In order to secure the number of cases required for statistical analysis in the working sample, additional participants who returned to work were recruited, and 51 workers completed a telephone survey in 2019. The final sample used for analysis was 323, 200 in the not-working group and 123 in the working group (pooled data set of 72 face-to-face survey data and 51 telephone survey data).

**Fig. 1:**
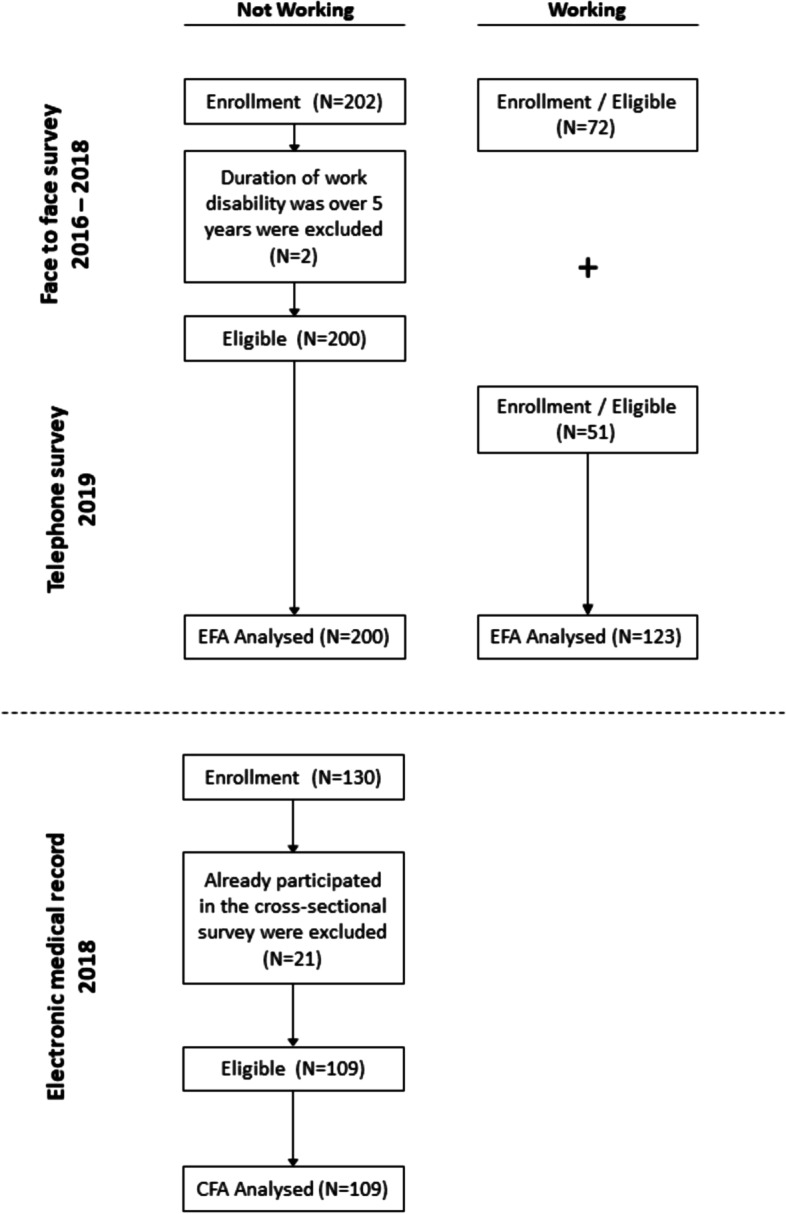
RrTW flow chart

The retrospective medical record data from patients’ FCE data were also collected to analyze the confirmatory factors. Since the research team had started to survey the RRTW scale, the scale was introduced in a pilot manner to the FCE procedure in KCOMWEL hospital. The patients who needed physical functional assessment results ahead of returning to work should complete the RRTW scale A-for not working with physical function assessment. As of December 2018, 130 workers had completed the RRTW scale at the KCOMWEL Incheon Hospital; 109 of these were included in CFA; 21 who had already participated in the cross-sectional survey were excluded (Fig. 1).

Insert Fig. 1 about here.

### Instruments

The RRTW scale measures psychosocial readiness in individuals preparing to return to work (Scale A) and maintenance levels in individuals who have already returned to work (Scale B). Scale A consists of 13 items on a 5-point Likert scale. The original scale demonstrated adequate reliability statistics (Cronbach’s alpha = 0.65 to 0.82) [8]. Scale B comprises nine items measured on a 5-point Likert scale with reasonable reliability ranging from 0.65–0.86.

Previous studies identified associations of RRTW subscales with related constructs: pain, fear-avoidance, general health, depressive symptoms [8, 9], job satisfaction [14], and perceived job performance [15]. Based on this, other variables and measurements were constructed as follows:

Pain was assessed using the Von Korff Pain Scale [16], where one item assessed current pain on a scale ranging from 0 (no pain) to 10 (the worst pain possible).

The Fear-Avoidance Beliefs Questionnaire (FABQ) comprised 16 items rated on a 7-point Likert scale. A fear-avoidance score used for this study consisted of the total score summed from 11 items, assessing two types of fear-avoidance beliefs related to physical activity and work. Internal consistency for the two subscales was 0.77 and 0.88, respectively, in a previous study [17] and 0.72 and 0.84 in this study.

General health was measured with the Short Form-12 (SF-12) [18], which assesses health on a scale from 0 (bad) to 100 (good), and includes both physical and mental health sub-scores (PCS 12 and MCS 12).

The Korean version of the Patient Health Questionnaire (PHQ-9) was used to assess depressive symptoms, adopting 9 items on a 4-point Likert scale [19, 20]. Internal reliability for this questionnaire was high in both a previous study and this study (Cronbach’s alpha = 0.89).

Satisfaction with working life comprised seven items (wage or income level, employment stability, work content, work environment, work time, potential for personal development, and communication and interpersonal relationships in the workplace) scored on a 5-point Likert scale (very dissatisfied to very satisfied; Cronbach’s alpha = 0.86).

Self-perceived work performance was measured with a single item, “If your work performance immediately before the industrial accident was 10 points, how would you rate your current work performance?” with responses ranging from 0 to 10.

### Statistical analysis

To test the RRTW scale’s structural validity, EFA and CFA were performed. Structural validity is the degree to which the scores of a Health-Related Patient-Reported Outcome (HR-PRO) instrument are an adequate reflection of the dimensionality of the construct to be measured, as explained in the “Consensus-based Standards for the selection of health measurement instruments risk of bias checklist” (COSMIN) [21]. We applied EFA because the RRTW scale had not been previously developed and administered to a Korean sample, the subscale structure was uncertain in Korean context, and also because previous studies of other countries [9, 22] have not shown consistent factor structure. Separate EFA was used for the not working and working samples, while CFA was applied only for the not working sample owing to the small sample size of working group despite two data collections. The second not-working group sample was insufficient in numbers but barely passed the sample size as rules-of-thumb such as 5:1 [23], 5:1 to 10: 1 [24] (i.e., 5 or 10 participants per variable) recommended. The sample strength of this study is similar to the previous studies [9]. Perhaps patients with work-related musculoskeletal injury in hospitals is a difficult topic to investigate; previous studies were also limited by the sample size.

In EFA, the maximum likelihood estimation [25] was used for factor extraction accounting for the sample characteristics, and direct oblimin rotation [26] was applied. Subsequently, the Kaiser-Meyer-Olkin (KMO) measure of sample adequacy (> 0.6) [23] and Bartlett’s sphericity test (< 0.05) [27] were calculated to examine suitability for factor analysis. Items with low factor loading (≤ 0.3) [28] or cross-loading (factor loading ≥0.3 in two or more factors) were deleted [28, 29]. CFA was performed using the maximum likelihood estimation using the following fit indices: Bentler’s comparative fit index (CFI), the Tucker-Lewis index (TLI), root mean square error of approximation (RMSEA), and the standardized root mean square residual (SRMR). CFI and TLI greater than 0.90 and RMSEA and SRMR less than 0.08 were considered reasonable fit [25, 30].

Criterion validity estimates the degree to which the instrument correlates to a relevant outcome. To confirm the concurrent validity of the RRTW scale, both a multidimensional and an allocation approach were used following Franche et al.’s [8] suggestion. In the multidimensional approach, Pearson correlation was calculated between each RRTW dimension and the relevant variables. In the allocation approach, Kruskal-Wallis non-parametric analysis of variance (ANOVA) and Mann-Whitney U tests were used to explore the differences between the RRTW stage groups on the health-relevant or occupational variables. Thereafter, a Mann-Whitney U test with Bonferroni correction was used for post-hoc analysis. For the allocation approach, we first categorized participants into one of the stages by averaging corresponding items for one specific stage and selecting the stage with the highest average score. If participants had the same score for more than one stage, the least advanced stage was chosen. If four stages had the same score, the participant was excluded. The same score was observed for multiple stages in 48 of 309 participants in the not-working sample (two stages: 40 workers, three stages: 8 workers). The same score was observed for multiple stages in four of 123 workers in the working sample.

Internal consistency, a measure of reliability, is the degree of interrelatedness among the items [21]. Cronbach’s α was calculated for all the subscales.

IBM SPSS Statistics, version 25.0, was used to perform descriptive statistics; the Mann-Whitney U test, the Kruskal-Wallis test, and EFA; Amos 20 (IBM; New York, United States) were used for CFA.

## Results

### Participant characteristics

A total of 309 participants comprised the not-working sample, and 123 participants were the working sample. To verify the representativeness of the sample, the data were compared with a representative dataset of workers involved in industrial accidents in South Korea in the First Wave Elementary Analysis Report on Workers’ Compensation and Welfare Insurance Panel Research from the Korea Labor Welfare Research Institute. Of the lost-time claimants in South Korea, 79.7% were men, 20.3% were women; 22.5% had completed middle school or less, 50.6% high school, 26.9% college or above; 37.9% worked in manufacturing, 10.8% wholesale/retail/accommodation/food, 20.7% construction, and 30.6% other fields (Table 1).

**Table 1:** Socio-demographic characteristics

	Not working sample (*n* = 309)		Working sample (n = 123)	
	N	%	N	%
Gender				
Male	268	86.7	107	87.0
Female	41	13.3	16	13.0
Age (Mean, SD)	48.5	(10.2)	46.0	(9.8)
Duration of Work Disability (Median / Range: Month)	10.3	(0–58)	23	(7–73)
Injured area of the body (more than one)				
Spine	154	41.7	44	35.8
Upper	94	25.5	38	30.9
Lower	121	32.8	64	52.0
Industrial Sector				
Manufacturing	128	41.4	52	42.3
Service	59	19.1	36	29.3
Construction	93	30.1	17	13.8
Others	29	9.4	18	14.6
Job Category				
Managers/professionals/clerks	40	12.9	35	28.5
Service/Sales workers	22	7.1	47	38.2
Craft workers/ Plant, machine operators/ assemblers	153	49.5	24	19.5
Elementary workers	92	29.8	17	13.8
Others	2	0.6	0	0.0
Type of Return to work				
pre-injury work	–	–	86	69.9
new work	–	–	37	30.1

### Exploratory and confirmatory factor analysis

#### RRTW scale a: for not-working group

##### Exploratory factor analysis

The KMO statistic was 0.68, and Bartlett’s test chi-square was 625.57 (*p* < .001), demonstrating suitability for EFA. EFA showed that using the original 13 items of the RRTW Scale A (*n* = 200) revealed a four-factor model: the precontemplation dimension (items a1, a2, and a13); the contemplation dimension (items a9, a11, and a12); the prepared for action: self-evaluative dimension (items a4, a7, and a8); and the prepared for action: behavioral dimension (items a3, a5, and a6). Item a10 was deleted because the factor loading was ≤0.3. The total variance explained by the final four-factor model was 48.18% (Table 2).

**Table 2:** Factor structure of the Readiness for Return-to-Work (RRTW) Scale

**Scale A (*N*** **= 200, for not work group)**		**Mean**	**SD**	**PC** **(α = 0.77)**	**C** **(α = 0.74)**	**PAS** **(α = 0.62)**	**PAB** **(α = 0.60)**
2.	As far as you’re concerned, there is no point in thinking about returning to work (PC)	1.83	1.31	**0.87**	−0.01	−0.07	0.11
1.	You don’t think you will ever be able to go back to work (PC)	2.04	1.40	**0.66**	0.17	−0.20	0.00
13.	As far as you are concerned, you don’t need to go back to work ever (PC)	1.44	1.02	**0.66**	−0.13	0.17	−0.20
11.	You wish you had more ideas about how to get back to work (C)	3.82	1.39	0.09	**0.89**	0.11	−0.19
12.	You would like to have some advice about how to go back to work (C)	3.51	1.51	0.07	**0.83**	−0.10	0.06
9.	You have been wondering if there is something you could do to return to work (C)	3.22	1.37	−0.10	**0.43**	−0.02	0.12
4.	Physically, you are starting to feel ready to go back to work (PAS)	2.74	1.22	−0.07	0.10	**0.80**	0.00
8.	You have found strategies to make your work manageable so you can return to work (PAS)	2.96	1.34	0.11	−0.07	**0.45**	0.17
7.	You are not ready to go back to work^a^ (PAS)	2.97	1.34	0.05	−0.08	**0.42**	0.09
6.	You are getting help from others to return to work (PAB)	3.32	1.56	0.02	−0.06	−0.01	**0.69**
5.	You have been increasing your activities at home to build up your strength to go back to work (PAB)	3.92	1.22	0.00	0.09	0.11	**0.49**
3.	You are actively doing things now to get back to work (PAB)	2.72	1.66	0.08	0.00	0.18	**0.36**
**Scale B (N = 123, for work group)**		**Mean**	**SD**	**PM** **(α = 0.58)**	**UM** **(α = 0.81)**		
4.	You found strategies to make your work manageable so you can stay at work (PM)	3.76	1.13	**0.92**	−0.17		
3.	You are taking steps to prevent having to go off work again due to your injury (PM)	3.59	1.34	**0.67**	0.12		
8.	You are back at work, and it is going well^a^ (UM)	3.97	1.10	**0.67**	−0.14		
2.	You learned different ways to cope with your pain so that you can stay at work (PM)	3.43	1.36	**0.64**	0.09		
1.	You are doing everything you can to stay at work (PM)	4.28	1.00	**0.56**	−0.04		
7.	You still find yourself struggling to stay at work due to the effects of your injury (UM)	4.04	1.21	0.25	**0.81**		
6.	You worry about having to stop working again due to your injury (UM)	2.87	1.50	−0.25	**0.49**		
9.	You feel you may need help in order to stay at work (UM)	3.19	1.26	−0.08	**0.46**		

^a^ item reversed

##### Confirmatory factor analysis

To test the fit of the RRTW model derived through EFA, CFA was performed on the second sample reviewing participants’ medical records regarding return to work or not (*n* = 109). The goodness of fit indices was: CFI = 0.92, TLI = 0.89, RMSEA = 0.07, and SRMR = 0.07, indicating that good model fit.

##### Reliability

Cronbach’s alpha was 0.77 for PC, 0.74 for C, 0.62 for PA, and 0.60 for PAB.

#### RRTW Scale B: For Working group.

##### Exploratory factor analysis

The KMO statistic was 0.73, and Bartlett’s test chi-square was 324.34 (*p* < .001), suggesting that the sample was suitable for EFA of RRTW Scale B (*n* = 123). Item b5 was deleted for cross-loading because it showed a factor loading of at least 0.3 in both the UM and PM domains. When the other items were subsequently re-analyzed, the UM subscale included items b6, b7, and b9, and the PM subscale included items b1, b2, b3, b4, and b8. Item b8 was related to the UM stage in the original scale, whereas it was more closely related to the PM stage items in the Korean adaptation. Factor analysis was performed again without reversing the question. The total variance explained by the two-factor model was 47.53% (Table 2).

##### Reliability

The internal consistency was analyzed for all subscales; Cronbach’s alpha was 0.58 for UM and 0.81 for PM.

### Concurrent validity

#### Multidimensional approach

We investigated the correlations of each dimension of the RRTW Scale A and B with relevant constructs to test concurrent validity (Table 3).

**Table 3:** Relationship between RRTW dimensions and health and occupational factors

**Item A**	**PC**	**C**	**PAS**		**PAB**	
Current pain	0.14	0.08	−0.18*		− 0.09	
Fear-avoidance- physical activity	0.27***	−0.00	− 0.26***		− 0.12	
Fear-avoidance- work	0.28***	0.24**	−0.51***		−0.21**	
Depression	0.35***	0.09	−0.33***		−0.20**	
General health-physical	−0.01	− 0.04	0.19**		0.12	
General health-mental	−0.26***	− 0.09	0.33***		0.12	
**Item B**	**UM**	**PM**				
Satisfaction with working life	−0.38***	0.42***				
Self-perceived work performance	−0.12	0.17				
**Constructs**	**PC**	**C**	**PAS**	**PAB**	**UM**	**PM**
Current pain	0.14	0.08	− 0.18*	− 0.09	0.29*	−0.03
Fear-avoidance- physical activity	0.27***	−0.00	− 0.26***	− 0.12	0.33**	− 0.10
Fear-avoidance- work	0.28***	0.24**	− 0.51***	− 0.21**	0.44***	−0.34**
Depression	0.35***	0.09	−0.33***	− 0.20**	0.40***	0.16
General health-physical	−0.01	− 0.04	0.19**	0.12	−0.39**	−0.39**
General health-mental	−0.26***	−0.09	0.33***	0.12	−0.44***	− 0.31**
Satisfaction with working life					−0.38***	0.42***
Self-perceived work performance					−0.12	0.17

*p < .05, **p < .01, ***p < .001

##### RRTW scale a: not-working group

The PC dimension showed positive correlations with fear-avoidance-physical activity (*r* = 0.27, *p* < .001), fear-avoidance-work (*r* = 0.28, *p* < .001), and depression (*r* = 0.35, *p* < .001); in contrast, it showed a negative correlation with general health-mental (*r* = − 0.26, *p* < .001). The C dimension showed a positive correlation with fear-avoidance-work (*r* = 0.24, *p* < .001). The PAS dimension showed negative correlations with current pain (*r* = − 0.18, *p* < 0.05), fear-avoidance-physical activity (*r* = − 0.26, *p* < .001), fear-avoidance-work (*r* = − 0.51, *p* < .001), and depression (*r* = − 0.33, *p* < .001), and showed positive correlations with general health-physical (*r* = 0.19, *p* < 0.05) and general health-mental (*r* = 0.33, *p* < .001). The PAB dimension showed negative correlations with FABQ work (*r* = − 0.21, *p* < .001), and depression (*r* = − 0.20, *p* < 0.05).

##### RRTW scale B: working group

The UM dimension was positively correlated with current pain (*r* = 0.29, *p* < 0.05), fear-avoidance-physical activity (*r* = 0.33, *p* < 0.01), fear-avoidance-work (*r* = 0.44, *p* < 0.001), and depression (*r* = 0.40, *p* < 0.001), whereas it was negatively correlated with general health-physical (*r* = − 0.39, *p* < 0.01), general health-mental (*r* = − 0.44, *p* < 0.001), and satisfaction with working life (*r* = − 0.38, *p* < 0.001). PM dimension was negatively correlated with fear-avoidance-work (*r* = − 0.34, *p* < 0.01), general health-physical (*r* = − 0.39, *p* < 0.01), general health-mental (*r* = − 0.31, *p* < 0.01), and positively correlated with satisfaction with working life (*r* = 0.42, *p* < 0.001).

##### Allocation approach.

We classified participants into one of six stages—four for the not-working sample and two for the working sample—based on their scores of readiness dimension and examined the differences in relevant constructs between RRTW stages to examine concurrent validity (Table 4).

**Table 4:** Differences in health and occupational factors between RRTW for not working and working group stages

Item A	PC (*n* = 19, 9.5%)		C (*n* = 95, 47.5%)		PAS (*n* = 33, 16.5%)		PAB (*n* = 53, 26.5%)		*x*^2^	Post-hoc
	M	SD	M	SD	M	SD	M	SD		
Current pain	5.63	2.14	5.60	1.75	4.94	2.22	4.85	2.09	7.64	–
Fear-avoidance-physical activity	20.74	4.04	15.87	5.15	14.06	4.97	15.79	5.87	19.49***	PC > C, PAS, PAB
Fear-avoidance-work	37.00	6.45	32.56	7.85	24.30	8.38	29.34	9.34	36.39***	PAB > PAS C > PAS PC > PAS, PAB
Depression	13.00	6.36	10.00	6.20	5.30	4.20	8.06	6.17	24.98***	C > PAS PC > PAS, PAB
General health-physical	36.72	8.59	35.89	7.39	38.15	6.53	36.10	8.24	2.76	–
General health-mental	36.38	10.47	42.58	11.06	48.52	8.88	44.29	12.57	13.28**	PC < PAB
Item B	UM (*n* = 49, 39.8%)		PM (*n* = 74, 60.2%)		Mann-Whitney U					
	M	SD	M	SD						
Satisfaction with working life	3.06	0.68	3.57	0.71	1065.50***					
Self-perceived work performance	6.29	2.11	9.23	0.73	929.50***					
**Constructs**	Not working group				Working group		*x*^2^ or Mann-Whitney U		Post-hoc	
	PC (9.5%)	C (47.5%)	PAS (16.5%)	PAB (26.5%)	UM (44.4%)	PM (55.6%)				
Current pain	5.63	5.60	4.94	4.85	5.19	4.85	9.23			
Fear-avoidance-physical activity	20.74	15.87	14.06	15.79	14.97	13.13	27.71***		PC > C,PAS,PAB, UM, PM	
Fear-avoidance-work	37.00	32.56	24.30	29.34	28.59	21.10	69.12***		PC > PAS, PAB, UM, PM C > PAS, UM, PM PAB > PM	
Depression	13.00	10.00	5.30	8.06	7.47	2.60	64.55***		PM < PC, C, PAB, UM PAS < PC, C UM < PC	
General health-physical	36.72	35.89	38.15	36.10	41.23	46.01	45.84***		PM > PC, C, PAS, PAB UM > C	
General health-mental	36.38	42.58	48.52	44.29	46.10	53.58	42.32***		PM > PC,C,PAB, UM PAS > PC	
Satisfaction with working life					3.06	3.57	1065.50 ***			
Self-perceived work performance					6.29	9.23	929.50 ***			

*p < .05, **p < .01, ***p < .001

Kruskal-Wallis non-parametric ANOVA results showed significant differences between RRTW stages for all the variables except current pain (x^2^ =9.23, *p* = 0.10), fear-avoidance of physical activity (x^2^ =27.71, *p* < 0.001), fear-avoidance of work (x^2^ =69.12, *p* < 0.001), depression (x^2^ =64.55, *p* < .001), general physical health (x^2^ =45.84, *p* = 0.43), and general mental health (x^2^ =42.32, *p* < .001) for both not-working and working participants. According to the results of post-hoc analysis, participants in the PC stage reported significantly the highest fear-avoidance of physical activity, and those in the PC and C stages showed high levels of fear-avoidance of work and depression. The PAS group tends to have lower fear-avoidance of work and depression and higher general physical and mental health than the PAS group; however, this result was not significant. The PM group reported the lowest depression with a low level of fear-avoidance and the highest general physical and mental health. The PM group reported significantly lower depression and higher general mental health than the UM group, with no significant difference in fear-avoidance of physical activity and work. On the work-related outcome, the PM group had significantly higher satisfaction with their working life (U = 1065.50, *p* < .001) and self-perceived work performance (U = 929.50, *p* < .001) than the UM group.

## Discussion

### Structural validity of RRTW

The structural validity of the Korean version of the RRTW scale was supported among workers who filed an accepted claim with the Korean worker’s compensation system following a work-related musculoskeletal injury. We identified four factors for the not-working group: PC, C, PAS, and PAB, and two factors for the working group: UM and PM, which are similar to those identified in the original scale by Franche et al. [8]

Compared with the original scale by Franche et al. [8], two items (item a10 and b5) were deleted from the scale, and one item (item b10) was loaded onto the other factor. Item a10 (“You have a date for your first day back at work”) was deleted from the PAS dimension because of the low factor loading, which could be explained by the Korean socio-cultural context and the fact that, in general, Korean workers covered by worker’s compensation showed a low rate of returning to their pre-injury job (42.5% in 2019). Moreover, the mean duration of work disability in our sample was seven months, indicating high severity and a high number of patients requiring long-term care, making it difficult to specify an RTW date during treatment. Item b5 was deleted because it showed cross-loading in both the UM and PM factors. Item b8 was found to be related to the PM dimension rather than the UM dimension. The same factor structure was derived in this study as in the original scale and by Park et al. [9], while it differed greatly from that of Braathen et al. [10].

The factor structure derived in this study was similar to that of Park et al.’s study [9] but differed greatly from that of Braathen et al.’s [10]. This difference may be related to the duration of work disability in the sample of each study. Park et al. [9] showed a three-factor structure that excluded the PC stage, and they deleted item a10, as in our study. The authors suggested differences in the sample characteristics with the original scale [9] as the reason for this discrepancy. Disability duration was an average of 188 days (about six months) in Park et al.’s [9] study and longer by one month in Franche et al.’s study [8]. The ratio of patients in the PC stage was relatively low in this study, at around 10%, which may be for the same reason as that of Park et al.’s study. Braathen et al. derived two factors: inability and uncertainty, and most items in the PAS and PAB dimensions, the advanced stage in the original scale, were deleted [10]. This may be because the participants were in the early stages of rehabilitation, suggesting that, to obtain more precise results, measurements should be taken after completing the rehabilitation program [10].

Cronbach’s alpha was 0.77 for PC, 0.74 for C, 0.62 for PAS, 0.60 for scale A, 0.58 for UM, and 0.81 for PM of scale B. The internal consistency with Cronbach’s alpha was acceptable for the PC, C, and PM, based on a criterion of 0.7 [31], but was low for the PAS and PAB, at around 0.6, and the UM, below 0.6. The results of scale A were similar to that of the original scale, but those of scale B showed a clear difference from previous studies, which reported satisfactory internal consistency for the UM but not for the PM [8–10]. More validation research is needed to understand the work maintenance dimension, as indicated by Braathen et al. [10].

### Concurrent validity

Concurrent validity was satisfied based on the correlations between RRTW dimensions and relevant constructs and ANOVA by RRTW stage groups. In the multidimensional approach, the PC or C dimensions, which are lower stages of the RRTW, generally showed positive correlations with negative statuses, such as fear-avoidance of physical activity or work and depression, and showed negative correlations with positive statuses, such as general mental health. In contrast, the PAS and PAB dimensions, which are advanced stages of the RRTW, showed the reverse. In the allocation approach, participants in the advanced stage exhibited better health-related and work-related outcomes than those in the less advanced stage. Specifically, the PC or C group reported higher fear-avoidance and depression, with lower general physical and mental health, which indicated that they had not yet been prepared physically and mentally for returning to work. Unsurprisingly, the PM group in the most advanced stage showed the best physical and mental health among readiness groups and clearly distinguished from UM group.

From both the multidimensional and allocation approach, some interesting findings were identified. First, the PC or C dimension had no association with perceived pain and physical health. This may be because participants scoring low on the PC or C dimension had some degree of pain and impaired physical function; therefore, low variability was observed in perceived physical function and pain. It can be understood in the same context that there is no difference in current pain between readiness groups.

Second, the PAS dimension, compared with the PAB dimension, showed a noticeably stronger correlation with relevant constructs. Moreover, participants in the PAS stage exhibited better health-related outcomes than those in the PAB stage, which is the most advanced stage among the not-working sample. In Franche et al.’s study, the original scale’s physical function-related scales (SF-12 physical, functional disability status, current pain, fear-avoidance of work) were perceived to be higher in the PAS stage than the PAB stage overall [8]. Park et al. reported better physical and mental health in the PAS stage [9]. There are several possible explanations for this finding. First, participants in this study were on long-term sick leave following the work-related injury, which could obscure the differentiation between PAS and PAB. Park et al. also explained that these results might be affected by the sample characteristics and suggested that PAS and PAB may be mixed in terms of subacute or chronic musculoskeletal disorders [9]. When they participate in intensive rehabilitation or work-hardening programs for several months, patients achieve the highest levels of confidence in RTW in the PAS stage. However, after this intense treatment, the availability for further rehabilitation on an outpatient basis decreases, and patients have to find other resources or help from other people to manage their physical health, which may lead to worse physical and mental outcomes in the PAB stage. Furthermore, the rate of returning to pre-injury jobs is low in South Korea, where the employer plays a lead role in the RTW process rather than the worker or medical personnel. Patients who cannot return to their pre-injury job and engage in job searching are likely to be in the PAB stage and may show increased mental health issues such as anxiety or depression. However, further studies are needed to investigate the differences between the PAB and PAS groups, and more information is required with regard to the use of the RRTW scale in a clinical setting.

Third, the UM dimension was the most correlated dimension positively with current pain, fear-avoidance of physical activity and work, and depression, and negatively with general physical and mental health, even stronger than the PC dimension. There were significant improvements compared with the least advanced PC or C group, but not better than PAS or PAB group. These results are similar to those in the original scale [8]. As Franche et al. [8] pointed out, it can be explained that maintenance of RTW itself is challenging to those who once returned to work. Even after returning to work following a work-related injury, most participants continued to experience pain and fear avoidance of physical activity. Moreover, uncertainty about re-injury or job maintenance can worsen their depressive symptoms and health condition, further lowering satisfaction with working life and work performance. The finding that the PM dimension had a significant association with work-related constructs, including fear-avoidance of work and satisfaction with working life, compared with UM dimension, seems to indicate that work burden and workplace factors act as an obstacle to proactive maintenance. As Franche et al. [8] pointed out, further research is needed to investigate the factors that determine the two groups. Moreover, interventions targeting workers in UM stage to help them re-adapt and stay in the workplace after work disability are warranted.

### Strengths and weaknesses

The construct and concurrent validity of the scale were verified for samples with a wide variety of injuries, levels of severity, and duration of work disability. Therefore, the scale has been adopted as an official evaluation tool for all the patients with musculoskeletal injuries in nine Workers’ Compensation Hospitals in Korea; further, validation may be possible in the near future.

However, some limitations should be addressed. First, a small sample size limited the statistical power of the CFA and the examination of the differences in relevant constructs between the RRTW stages. Although the guidelines for the minimum sample size needed for factor analysis varies, the non-working sample size (*n* = 109) for performing CFA in this study was insufficient. This is because it is difficult to recruit survey participants concerning industrial accidents; similarly, most previous studies on RRTW construct validity tests (see Additional File 1) also had small samples of less than 200. Furthermore, the small number of cases in the PC stage limited our interpretation. Despite the limitations of the statistical results due to the small sample size, this scale will be useful to assess patients with work-related musculoskeletal injuries who are preparing to return to work. It is necessary to continue to monitor how the RRTW scale, as a screening tool, predicts future work participation and to further develop a tailored intervention program according to the RRTW stage. In the future, through multi-center research, a more detailed interpretation may be possible by increasing the analyzed target numbers. Second, this study employed a cross-sectional design, which prevented the detection of changes to the RRTW stage over time. Therefore, follow-up studies are underway to observe the development among these patients.

## Conclusion

This study analyzed the internal consistency reliability and structural and concurrent validity of the Korean version of the RRTW scale, which aims to assess readiness to RTW and associated psychosocial variables. Although the validation process resulted in small changes to certain items, RTW readiness, and maintenance levels matched those of the original scale, suggesting that the Korean scale is suitable for use in research and clinical settings.

## Supplementary Information

**Additional file 1.**

## Data Availability

The datasets generated during and/or analyzed during the current study are available from the corresponding author on reasonable request.

## Abbreviations

RTW: return to work:
RRTW: readiness for return to work
EFA: exploratory factor analysis
CFA: confirmatory factor analysis
MSK: musculoskeletal disorders
PC: pre-contemplation
C: contemplation,
PAS: preparation for action-self-evaluation
PAB: preparation for action-behavioral
UM: uncertain maintenance
PM: proactive maintenance
FCE: Functional Capacity Evaluation
KCOMWEL: Korea Worker’s Compensation & Welfare Service
FAB-Q: Fear-Avoidance Beliefs Questionnaire
PHQ-9: Patient Health Questionnaire
KMO: Kaiser-Meyer-Olkin
CFI: comparative fit index
TLI: Tucker-Lewis index
RMSEA: root mean square error of approximation
SRMR: standardized root mean square residual
ANOVA: Analysis of variance
