# Supplementary material for: Psychometric properties of the readiness for return to work scale in occupational rehabilitation in South Korea
Source: BMC Public Health. 2023 Jan 6;23:39. doi: 10.1186/s12889-022-14948-2 (PMC9817403; doi:10.1186/s12889-022-14948-2)
Supplement: Supplementary file 1 — Additional file 1. [file 12889_2022_14948_MOESM1_ESM.docx]

**Supplementary Table 1** Summary of previous studies on the verification of the RRTW (Return to Work) scale

| **Study** | **Country** | **Participants** | **Method** | **Not working** | | | **Working** | | | **Result of Analysis** |
| --- | --- | --- | --- | --- | --- | --- | --- | --- | --- | --- |
|  |  |  |  | **Participants** | **Stage** | **Fators - item no.** | **Participants** | **Stage** | **Fators - item no.** |  |
| Franche et al. (2007) | Canada | - 632 lost–time claimants with work–related back or upper extremity MSK disorders  - Absent from work for a minimum of 5 days within the first 14 calendar days post–injury (based on self–report), and were at least 15 years old at the time of their injury. | EFA | 194 participants | 4 stages | PC: 1, 2, 13  C: 9, 11, 12  PAS: 4, 7, 8, 10  PAB: 3, 5, 6 | 166 participants | 2 stages | UM: 1, 2, 3, 4  PM: 5, 6, 7, 8, 9 | RRTW scale A  - 4 stage solution explained 60% of the variance.  - Cronbach’s alpha:  PM: 0.65, C: 0.69, PAS: 0.75, PAB: 0.67  RRTW scale B  - 2 stage solution explained 58% of the variance.  - Cronbach’s alpha:  UM: 0.82, PM: 0.67 |
|  |  |  | CFA | 150 participants |  |  | 167 participants |  |  | RRTW scale A  - CFI=0.92, X²=90.6, df=59, X²/df=1.54, Bollen IFI = .92, RMSEA = .06, NNFI=0.90  RRTW scale B  - CFI = .90, X²=57.9, df=26, X²/df= 2.2, Bollen IFI = .90, RMSEA = .08, NNFI=0.86  - Considering the Lagrange Multiplier Test, an error correlation was found between the items #6 and #9. By adding this error item correlation between the two items (re9e6), all fit indices were satisfactory  => CFI=.94, 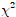 =45.2, df=25, 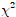/df=1.81, Bollen IFI=.94, RMSEA=.07, NNFI=.91 |
| Braathen et al. (2013) | Norway | -193 persons  -Evaluating a 5-day inpatient occupational rehabilitation program | EFA | 124 participants | 2 stages | Inability: 1, 2, 4, 5, 13  Uncertainty: 10, 11, 12 | 60 participants | 2 stages | UM: 1, 3, 5, 6  PM: 2, 4, 9 | RRTW scale A  - 2 stage solution explained 64.5 % of the variance.  - 3, 6, 7, 8, 9 drop > 0.3  - Cronbach’s alpha:  Uncertainty: 0.81, inability: 0.72  RRTW scale B  - 2 stage solution explained 55.1% of the variance.  - 8 drop > 0.2  - Cronbach’s alpha:  UM: 0.75, PM: 0.59 |
| Park et al. (2018) | Canada | - 389 claimants  - MSK disorders (Disability duration average 188 days) | EFA | -109 participants | 3 stages | C: a9, a11, a12  PAS: a4, a7, a8  PAB: a3, a5, a6 | 114 participants | 2 stages | UM: b1, b2, b3, b4  PM: b5, b6, b7, b8, b9 | RRTW scale A  - a4, a7 related to Prepared for Action-Self-evaluative.  - a10 drop: <0.3  - 3 stage solution explained 65.8% of the variance.  - Cronbach’s alpha:  C: 0.72, PAS: 0.69, PAB: 0.72  RRTW scale B  - 2 stage solution explained 47.5% of the variance.  - Cronbach’s alpha:  UM: 0.76, PM: 0.59 |
|  |  |  | CFA | - |  |  | 111 participants |  |  | RRTW scale B items only.  Fit of the model: acceptable  CFI=0.92, X²=38.82, df=27, X²/df=1.44, RMSEA=0.04 |
| Stapel-feldt et al. (2018) | Denmark | - 374 sickness beneficiaries from a municipal employment agency and 97 out-patients from three hospital wards in the Central Region Denmark  - After eight sick leave weeks | EFA | 381 participants | 4 stages | PC: 1, 2, 13  C: 9, 11, 12  PAS: 4, 7, 8, 10  PAB: 3, 5, 6 | 90 participants | 2 stages | UM: 5, 6, 7, 8, 9  PM: 1, 2, 3, 4 | RRTW scale A  - Cronbach’s alpha:  PC: 0.71 C: 0.44, PAS: 0.78, PAB: 0.54  RRTW scale B  - Cronbach’s alpha:  UM: 0.79, PM: 0.81 |
|  |  |  | CFA | 373 participants |  |  | Not implemented  (Sample was too small) |  |  | RRTW scale A  -X^2^=397.1, p<0.001, CFI= 0.934, TLI=0.913, RMSEA=0.124, SRMR=0.100 |
|  |  |  | Test-retest reliability | 84 participants |  |  | 30 participants |  |  | RRTW scale A (ICC)  PC: 0.77, C: 0.37, PAS: 0.47, PAB: 0.47  RRTW scale B (ICC)  UM: 0.71, PM: 0.44 |

PC: Pre-contemplation, C: Contemplation, PAS: Preparation for action-self-evaluation, PAB: Preparation for action-behavioral, UM: Uncertain Maintenance, PM: Proactive Maintenance
